# Alkalinization in the Isolated and Perfused Anterior Midgut of the Larval Mosquito, *Aedes aegypti*

**DOI:** 10.1673/031.008.4601

**Published:** 2008-06-04

**Authors:** Horst Onken, Stacia B. Moffett, David F. Moffett

**Affiliations:** ^1^ Department of Biological Sciences, Wagner College, Staten Island, NY 10301, USA; ^2^ School of Biological Sciences, Washington State University, Pullman, WA 99164–4236, USA

**Keywords:** amiloride, concanamycin, insect, larva, m-cresol purple, methazolamide, midgut, serotonin, transepithelial voltage

## Abstract

In the present study, isolated midguts of larval *Aedes aegypti* L. (Diptera: Culicidae) were mounted on perfusion pipettes and bathed in high buffer mosquito saline. With low buffer perfusion saline, containing m-cresol purple, transepithelial voltage was monitored and luminal alkalinization became visible through color changes of m-cresol purple after perfusion stop. Lumen negative voltage and alkalinization depended on metabolic energy and were stimulated in the presence of serotonin (0.2 µmol l^-1^). In some experiments a pH microelectrode in the lumen recorded pH values up to 10 within minutes after perfusion stop. The V-ATPase inhibitor concanamycin (50 µmol l^-1^) on the hemolymph side almost abolished V_te_ and inhibited luminal alkalinization. The carbonic anhydrase inhibitor, methazolamide (50 µmol l^-1^), on either the luminal or hemolymph-side, or the inhibitor of anion transport, DIDS (1 mmol l^-1^) on the luminal side, had no effect on V_te_ or alkalinization. Cl^-^ substitution in the lumen or on both sides of the tissue affected V_te_, but the color change of m-cresol purple was unchanged from control conditions. Hemolymph-side Na^+^ substitution or addition of the Na^+^/H^+^ exchange inhibitor, amiloride (200 µmol l^-1^), reduced V_te_ and luminal alkalinization. Luminal amiloride (200 µmol l^-1^) was without effects on V_te_ or alkalinization. High K^+^ (60 mmol l^-1^) in the lumen reduced V_te_ without affecting alkalinization. These results indicate that strong luminal alkalinization in isolated and perfused anterior midgut of larval *A. aegypti* depends on basolateral V-ATPase, but is apparently independent of carbonic anhydrase, apical Cl^-^/HCO_3_^-^ exchange or apical K^+^/2H^+^ antiport.

## Introduction

Strong alkalinization of up to pH 12 is a characteristic feature of midgut regions of many larvae of endopterygote insects, including members of the orders Coleoptera, Diptera, Trichoptera, and Lepidoptera (for references see Clark 1999). Insect midgut ion transport has been studied most intensively with lepidopteran larvae (for reviews see Dow 1986; Klein et al. 1996). The currently accepted hypothesis is based on the observation of K^+^/2H^+^ antiport in goblet membrane vesicles (Wieczorek et al. 1991; Azuma et al. 1995): According to this proposal, apical V-type H^+^ pumps energize electrogenie K^+^/2H^+^ exchangers in the apical membrane, resulting in active K^+^ secretion and strong alkalinization (for reviews see Lepier et al. 1994; Wieczorek et al. 1999).

Because larval mosquitoes drink the ambient medium (Clements 1992), midgut pH can be studied by addition of pH indicators to the water in which the animals are maintained. Midgut pH can then be determined by the indicator color in the transparent larvae. Strong alkalinization was observed in the anterior midgut [or “anterior stomach”, in the terminology of Clements (1992)] and pH values above 10 have been observed (Dadd 1975; Zhuang et al. 1999). It was also recognized that larvae were incapable of alkalinization after bafilomycin, a specific inhibitor of V-ATPases (Dröse and Altendorf 1997), had been added to the water (Zhuang et al. 1999). V-ATPase was indeed found to be highly expressed in the midgut of larval mosquitoes (Filippova et al. 1998), but was, unlike in lepidopteran larvae, localized with antibodies in the basal membrane of the epithelial cells of the anterior midgut (Zhuang et al. 1999). This basal localization of V-ATPase was later verified in experiments with V-ATPase inhibitors in “semi-intact” larval mosquitoes and with isolated larval midgut segments (Boudko et al. 2001a, Onken et al. 2004a, 2006). Because similar results were also obtained with isolated and perfused posterior midguts of larval *Drosophila melanogaster* (Shanbhag and Tripathi 2005), it could be assumed that basolateral localization of V-ATPase may be a feature of alkalinizing midgut epithelia of dipterans. Apart of bafilomycin also addition of inhibitors of carbonic anhydrase (acetazolamide, methazolamide) or of anion transporters (DIDS) to the ambient medium abolished or reduced the ability of intact larvae to alkalinize the anterior midgut (Boudko et al. 2001a; Corena et al. 2002).

Boudko et al. (2001a, b) introduced a technique that used “semi-intact” larvae in which the intact midgut was exposed by a slit in the cuticle and ion gradients (pH, Cl^-^) were measured at the hemolymph-side surface of the anterior midgut with ion sensitive microelectrodes. Ion gradients indicative for HCl efflux from the tissue to the hemolymph were abolished or reduced after application of the V-ATPase inhibitor, bafilomycin, inhibitors of carbonic anhydrase or DIDS. These results were summarized in a transport model where basal V-ATPase energizes apical Cl^-^/HCO_3_^-^ exchangers. The intracellular substrates of these transporters were proposed to be rapidly supplied through the action of carbonic anhydrase. Cl^-^ ions that enter the cells from the lumen via the exchangers were proposed to exit to the hemolymph through basal Cl^-^ channels. It was recognized, however, that this HCO_3_^-^ secretion alone can only explain luminal alkalinization up to pH values of about 8.5, and that alkalinization to higher pH values would need additional active and transepithelial absorption of H^+^ (or secretion of strong base).

Clark et al. (1999) measured the transepithelial voltage (V_te_) of isolated and perfused midgut segments. The initially very high V_te_ of anterior (lumen negative) and posterior (lumen positive) midgut segments rapidly declined after mounting and were only partly restored after addition of hemolymph-like, submicromolar concentrations of serotonin. In a subsequent study of the isolated anterior midgut (Clark et al. 2000), two different cell populations were discovered with microelectrodes and it was assumed that these two cell types could reflect the columnar and cuboidal cells observed microscopically (Zhuang et al. 1999). Altogether, the results were consistent with the assumption that the initially high, lumen negative V_te_ generated by the anterior midgut reflects HCO_3_^-^ secretion via one cell type and H^+^ absorption (or strong base secretion) via the other cell type. However, isolation and mounting of the tissue deactivates both cell populations and serotonin only reactivates the HCO_3_^-^ secreting cells. Onken et al. (2004a) measured V_te_ of the isolated and perfused anterior midgut in the presence of serotonin and analyzed the effects of a number of inhibitors and ion substitutions. As a result of this study the transport model by Boudko et al. (2001a; see above) was refined and Na^+^ -dependent and Na^+^ -independent modes of active HCO_3_- secretion were distinguished. Onken et al. (2004b) used a number of peptide hormones in the presence of serotonin in order to completely re-establish the initially high V_te_ and to reactivate both cell populations. However, the peptide hormones used in that study had either no effect or even reduced V_te_.

Already Dadd (1975) observed that the high pH in the anterior midgut of intact larvae was very unstable and “slight injury or even just handling” could result in loss of alkalinity. The results obtained with anterior midgut preparations (see above) suggested that strong alkalinization declines after isolation and mounting as in lepidopteran larvae (Clark et al. 1998). The objective of the present study was to verify this assumption, using a pH indicator in the luminal saline in order to monitor alkalinization in the isolated anterior midgut of larval *Aedes aegypti*. To our surprise we observed that the isolated anterior midgut is indeed capable of strong alkalinization in the presence of serotonin. Even more surprising, the results obtained indicate that strong alkalinization in the isolated tissue is independent of carbonic anhydrase or HCO_3_^-^ secretion via apical anion exchange.

## Materials and Methods

### Mosquitoes

*Aedes aegypti* (Vero Beach strain) eggs were provided by Dr. Marc Klowden (University of Idaho, Moscow, USA) from a continuously maintained colony. Eggs were hatched and larvae were maintained in a 1:1 mixture of tap water and deionized water at 26 °C and on a 16:8 L:D photoperiod. The water was replaced each morning, and the larvae were fed with ground Tetramin flakes (Tetrawerke, Melle, Germany). Fed fourth-instar larvae were used in all experiments.

### Solutions and chemicals

The basic NaCl saline used to perfuse the bath (hemolymph-side of the epithelium) was based on larval *Aedes* hemolymph composition (Edwards 1982a, b) and consisted of (in mmol l^-1^): NaCl, 42.5; KCl, 3.0; MgCl_2_, 0.6; CaCl_2_, 5.0; NaHCO_3_, 5.0; succinic acid, 5.0, malic acid, 5.0; L-proline, 5.0; L-glutamine, 9.1; L-histidine, 8.7; L-arginine, 3.3; dextrose, 10.0; Hepes, 25. The pH was adjusted to 7.0 with NaOH. In Na^+^-free saline, NaCl was substituted with N-methylglucamine. Instead of 5 mmol l^-1^ NaHCO_3_, this saline contained 3 mmol l^-1^ KHCO3 (no KCl). The pH was adjusted with HCl. In Cl^-^-free saline, gluconates (Na^+^, K^+^, Ca^2+^) or sulfate (Mg^2+^) substituted for the chlorides. The pH was adjusted with NaOH. The salines to perfuse the lumen of the tissue were identical to the above salines, but contained 0.04 % m-cresol purple (Aldrich, www.sigmaaldrich.com) and reduced buffer (0.25 mmol l^-1^ Hepes). In the high K^+^ saline the K^+^ concentration was increased to 60 mmol l-^1^ by replacing NaHCO_3_ with KHCO_3_ and part (35 mmol l^-1^) of NaCl with KCl (titration with KOH 20 mmol l^-1^). The above components were purchased from Sigma (www.sigmaaldrich.com), Fisher Scientific (www1.fishersci.com) or Mallinckrodt (www.mallinckrodt.com). Serotonin (Sigma) and dinitrophenol (DNP; Eastman Kodak Company, www.kodak.com) were directly dissolved in the saline. Amiloride (Sigma) was added to the saline from an aqueous stock solution of 10 mmol l^-1^. Concanamycin (Fluka, www.sigmaaldrich.com), methazolamide and 4,4′-diisothiocyanato-stilbene-2,2′-disulfonic acid (DIDS, both from Sigma) were added from stock solutions in dimethylsulfoxide (DMSO; Sigma). The primary solvent alone had no effect on voltage or alkalinization at the concentrations present in the experiments.

### Preparation, mounting, perfusion and measurement of the transepithelial voltage

Manufacture of perfusion pipettes as well as preparation, mounting and perfusion of anterior midguts was almost identical to those outlined in detail before (Onken et al. 2004a,b) and will be described only briefly here.

After killing of the larvae, the intestinal system was isolated and transferred to the bath of a perfusion chamber. The caeca, the hindgut and the Malpighian tubules were cut off and the posterior midgut was slipped onto an Lshaped perfusion pipette held by a micromanipulator (Brinkmann, www.brinkmann.com) until the tip of the pipette recorded the typical, high, lumen negative transepithelial voltage (V_te_). The preparations were tied in place with a fine human hair. In order to keep the preparation in the focus of the binocular microscope, the open anterior end of the midgut preparation was slipped onto a glass rod manufactured from a glass capillary pipette (20 µl; VWR, www.vwrsp.com) and held by a second micromanipulator. The bath (volume 100 µl) was gravity perfused (rate 15–30 ml h^-1^) with oxygenated salines. The perfusion pipette contained a polyethylene tubing (Intramedic PE 10; VWR) and was closed with a syringe needle. The syringe needle and the tubing were connected to two push-pull multi-speed syringe pumps (model 120; Stoelting, www.stoelting.com), allowing fast changes of the luminal perfusion (rate 30 µl h^-1^) between two different solutions. The transepithelial voltage (V_te_) was measured exactly as described before (Onken et al. 2004a,b). Only those preparations that showed the typical marked increase of V_te_ after application of serotonin (0.2 mmol l^-1^; cf. Clark et al. 1999) were used for data collection.

### Alkalinization

Alkalinization of the luminal perfusate was monitored after perfusion stop through the color changes of the pH indicator m-cresol purple (_p_K_1_ ∼ 2.0, _p_K_2_ = 8.32). Color changes were documented with a digital camera (Power-Shot S40; Canon, www.canon.com) before and at 5 minute time intervals after perfusion stop. All photographs were identically edited (cropped, adjustment of lighting) with iPhoto Express (Ulead Systems, www.ulead.com). In order to estimate the pH, polyethylene tubing with the approximate dimensions of the anterior midgut (Intramedic PE 10; ø_i_ = 0.28 mm, ø_o_ = 0.61 mm) was inserted into the bath and perfused with NaCl saline of different pH, containing 0.04 % m-cresol purple. The colors of these solutions were documented as described above and are shown in Figure 1. In some experiments, thymol blue (Sigma; _p_K_1_ ∼ 1.65, _p_K_2_ = 9.20) was used instead of m-cresol purple. In some experiments pH-sensitive microelectrodes were prepared (as described in detail in Chao et al. 1991) and replaced the glass rod inserted into the anterior end of the anterior midgut in order to measure the luminal pH. In preliminary experiments it was verified that the transepithelial voltage is not affected when low buffer saline with pH indicators was used as luminal perfusate.

**Figure 1.**
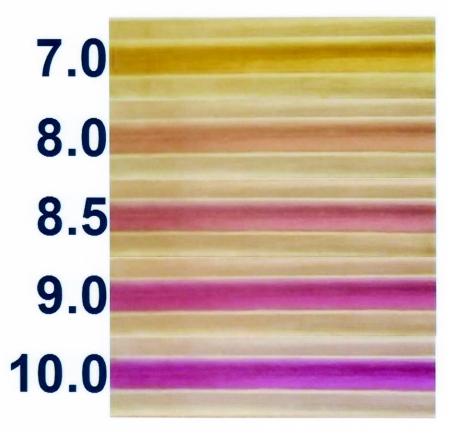
Photographs of low buffer salines containing 0.04 % m-cresol purple of indicated pH values perfused through PE10 tubing in the perfusion chamber used for the measurements with isolated and perfused anterior midguts of larval *Aedes aegypti*.

### Statistics

All data are presented as means ± standard error of the mean (S.E.M.). Differences between groups were tested, using Student's t-test. Significance was assumed at *P* < 0.05.

## Results

### Alkalinization of isolated and perfused anterior midgut preparations

When anterior midgut preparations were bathed in hemolymph-like NaCl saline and perfused with low buffer NaCl saline containing m-cresol purple, a mean transepithelial voltage (V_te_) of -12 ± 1 mV (± S.E.M., N = 50; lumen negative) was measured. After application of 0.2 µmol l^-1^ serotonin the mean lumen negative V_te_ increased to a more negative value of -45 ± 3 mV (± S.E.M., N = 50). These voltages were very similar to the results obtained previously with the same tissue (Clark et al. 1999; Onken et al. 2004a,b). In 9 experiments, the perfusion was stopped before and after application of serotonin and the ability of the tissue to alkalinize the luminal perfusate was evaluated. Figure 2 shows a typical V_te_ time-course of such an experiment. Under control conditions the lumen negative V_te_ slightly dropped to less negative values during the phase of luminal perfusion stop, whereas V_te_ markedly droped when the luminal perfusion was stopped in the presence of serotonin. After re-establishing luminal perfusion, V_te_ recovered to the value before perfusion stop. During the V_te_ decline after perfusion stop the color of m-cresol purple changed from yellow to purple, indicating that the luminal saline was alkalinized. In the absence of serotonin the color change of m-cresol purple was either absent or developed slowly. In all 9 experiments the color change was faster and more pronounced in the presence of serotonin. In Video 1, alkalinization in the presence of serotonin can be observed from luminal perfusion stop to re-start of the perfusion. The video clearly demonstrates that alkalinization began in the most anterior portion of the anterior midgut and then continued to spread in a posterior direction. In Figure 3, a representative example tissue is shown right after perfusion stop in control saline, 15 minutes after perfusion stop in control saline and 15 minutes after perfusion stop in saline containing serotonin. Eight experiments with the uncoupling reagent DNP were conducted in the presence of serotonin in order to verify that the generation of V_te_ and the alkalinization were based on active, ATP consuming transport processes. In all cases, DNP abolished V_te_ and alkalinization was never observed in the presence of DNP.

### Effects of the V-ATPase inhibitor concanamycin on V_te_ and alkalinization

With intact and semi-intact larvae it was observed that addition of bafilomycin, a specific blocker of V-ATPases (Dröse and Altendorf 1997), inhibited alkalinization in the anterior midgut and reduced the pH gradient on the hemolymph-side surface of the tissue (Zhuang et al. 1999; Boudko et al. 2001b). In order to verify this result with the isolated and perfused tissue another specific V-ATPase inhibitor, concanamycin (50 µmol l^-1^; Dröse and Altendorf 1997) was added to the serotonin containing saline in the bath. After incubation for 60 minutes the drug almost abolished the lumen negative V_te_ (from -41 ± 9 mV to -3 ± 1 mV; ± S.E.M., N = 5, P < 0.05) and alkalinization was markedly reduced (Figure 4).

**Video 1**

The video (3:35 minutes at four times accelerated speed) shows a typical experiment with an isolated and perfused anterior midgut of larval *A. aegypti*. Luminal solution: low buffer mosquito saline with m-cresol purple (0.04%). Hemolymph-side solution: high buffer mosquito saline with serotonin (0.2 µmol l^-1^). The posterior part of the preparation is tied with a human hair onto a perfusion pipette (left) and a glass rod inserted into the lumen (right) maintains the tissue in the focus of the microscope. Luminal perfusion was stopped at the beginning of the video. The color change of m-cresol purple that reflects alkalinization (from yellow to purple) begins at the anterior end of the midgut (right) and continues towards more posterior regions of the anterior midgut (left). The peristaltic muscular activity can also be easily observed (cf. Onken et al. 2004b). At 2:40 minutes, the perfusion pump is started again and the purple, alkaline solution is flushed from the lumen of the preparation. This video can be accessed at the following URI: http://digital.library.wisc.edu/1793/28226

### Effects of inhibition of carbonic anhydrase on V_te_ and alkalinization

In previous studies inhibitors of carbonic anhydrase (acetazolamide, methazolamide) markedly reduced alkalinization in the anterior midgut of intact larvae (Boudko et al. 2001a; Corena et al. 2002). Moreover, the drugs significantly reduced the gradients for H^+^ and Cl^-^ at the hemolymph-side surface of the anterior midgut of semiintact larvae that are indicative for HCl flux from the epithelial cells to the hemolymph (Boudko et al. 2001a). On the other hand, the transepithelial voltage of isolated and perfused anterior midgut preparations was not significantly affected by acetazolamide (Onken et al. 2004a). In the present study the effects of methazolamide (200 µmol l^-1^) on V_te_ and alkalinization in isolated and perfused anterior midgut preparations were monitored. Applying the drug to the hemolymph-side bath for 10 to 20 minutes had no significant effect on V_te_ (P > 0.05), and alkalinization was equally observed in the presence and in the absence of methazolamide (N = 5; see Figure 5). Similarly, no significant effects on V_te_ (P > 0.05) and alkalinization were observed when the lumen of the isolated anterior midgut was perfused for 10 to 15 minutes with methazolamide (N = 6; see Figure 6). In two experiments methazolamide was present on both sides of the epithelium, but again, no effects on V_te_ or alkalinization were observed.

### Effects of luminal DIDS and Cl^-^ substitutions on V_te_ and alkalinization

Boudko et al. (2001a) found that alkalinization in intact larvae was inhibited when DIDS was added to the medium. Moreover, the drug inhibited H^+^ and Cl^-^ gradients at the surface of the exposed anterior midgut of semiintact larvae. Onken et al. (2004a) observed that DIDS reduced lumen negative V_te_ to less negative values when it was applied to the hemolymph-side of the isolated tissue, and the drug caused an additional V_te_ decrease when it was present on both sides of the epithelium. In the present study, we perfused the lumen of the anterior midgut for 10 to 30 minutes with DIDS (1 mmol l^-1^, N = 5), but this manipulation did not significantly affect V_te_ (P > 0.05) and alkalinization was observed as in the controls before addition of the drug (see Figure 7). In the next series of experiments (N = 5), Cl^-^ was substituted in the lumen and then, in a second step, also in the bath. After on average 10 minutes of substitution of CF in the lumen, V_te_ significantly increased from -50 ± 5 mV to -63 ± 7 mV (N = 5, ± S.E.M., P < 0.05). Alkalinization was still observed as in the control before substitution of luminal Cl^-^. Subsequent additional substitution of Cl^-^ in the hemolymph-side bath for another 10 minutes significantly reduced the lumen negative voltage to -18 ± 5 mV (N = 5, ± S.E.M., P < 0.05), but alkalinization was observed even under these conditions (Figure 8).

**Figure 2.**
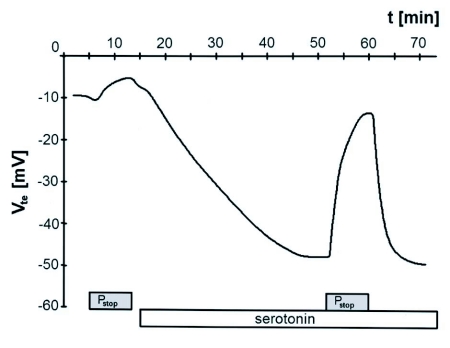
Representative time-course of the lumen negative transepithelial voltage (V_te_) of a preparation of the anterior midgut of larval (4^th^ instar) *Aedes aegypti*, showing the effects of serotonin and luminal perfusion stop.

### Effects of hemolymph-side Na^+^ substitution on V_te_ and alkalinization

Onken et al. (2004a) observed that hemolymph-side substitution of Na^+^ decreased lumen negative V_te_ to less negative values. On the basis of this finding, the authors proposed that part of transapical HCO_3_^-^ secretion may follow a Na^+^ -dependent mode via apical Na^+^/2–3 HCO_3_- symporters. DIDS insensitive Na^+^/HCO_3_-symporters are known (Boron 2001), although they seem to be electroneutral. In the present study we repeated the experiments with Na^+^ -free salines in the hemolymph-side bath, evaluating the capacity of alkalinization in addition to the effects on V_te_. As in the previous study (see V_te_ time-course in Onken et al. 2004a), substitution of hemolymph-side Na^+^ for approximately 30 minutes significantly reduced the lumen negative V_te_ from -46 ± 6 to -8 ± 1 mV (N = 9, ± S.E.M., P <0.05; see Figure 9). In addition, hemolymph-side Na^+^ substitution indeed abolished strong alkalinization (see Figure 9).

### Effects of hemolymph-side amiloride on V_te_ and alkalinization

Onken et al. (2004a) observed that addition of amiloride to the hemolymph-side bath decreased lumen negative V_te_ to less negative values, and the authors proposed that a basolateral Na^+^/H^+^ exchanger may contribute to proton absorption from the cells to the hemolymph. Na^+^ entering the cells via the exchanger could supply apical Na^+^/2^+^–-3 HCO_3_^-^ symporters with Na^+^. Hemolymph-side amiloride (200 µmol l^-1^), was used to evaluate the capacity of alkalinization in addition to the effects on V_te_. As in the previous study, hemolymph-side amiloride significantly reduced V_te_ from -52 ± 11 to -19 ± 4 mV (N = 7, ± S.E.M., P < 0.05; see Figure 10) within an average exposure time of 25 minutes. Moreover, the drug reduced alkalinization (see Figure 10).

**Figure 3.**
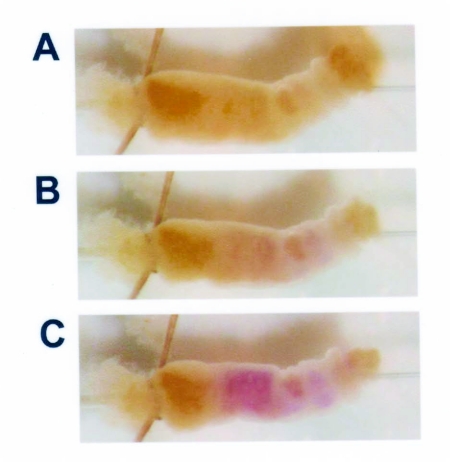
Photographs of a preparation of the anterior midgut of larval (4^th^ instar) *Aedes aegypti*. A) Control conditions directly after luminal perfusion stop. B) Control condition 15 minutes after perfusion stop. C) In the presence of serotonin (0.2 µmol l^-1^) 15 minutes after perfusion stop.

### Effects of luminal amiloride and high K^+^ on V_te_ and alkalinization

According to proposals for the midgut of larval *Manaduca sexta*, strong alkalinization could result from transapical, electrogenic K^+^/2H^+^ exchange energized by apical V-type ATPases (Wieczorek et al. 1991). In order to test the hypothesis that luminal alkalinization in larval mosquitoes is based on the basolateral V-ATPase energizing apical K^+^/2H^+^ antiport, 200 µmol l^-1^ amiloride was used as it was shown to reduce K^+^/2H^+^ antiport in *M. sexta* goblet membrane vesicles by 50 % (Wieczorek et al. 1991). However, addition of 200 µmol l^-1^ amiloride to the luminal perfusate for 10 to 30 minutes had no effects on V_te_ or alkalinization (see Figure 11). Amiloride is apparently not a very specific inhibitor of K^+^/2H^+^ antiporters, because a very high concentration (1 mmol l^-1^) was needed to abolish K^+^/2H^+^ antiport in membrane vesicles from *M. sexta* midgut (Wieczorek et al. 1991), and in the isolated midgut epithelium even 10 mmol l^-1^ was needed to affect the short circuit current (Schirrmanns and Zeiske 1994). Therefore, it could be that amiloride is not a good tool to identify K^+^/2H^+^ antiporters. In another series of experiments K^+^ was increased in the luminal saline to 60 mmol l^-1^ (at reduced Na^+^) to reduce or abolish the transapical K^+^ gradient. This manipulation should at least reduce transapical K^+^/2H^+^ antiport. In five experiments high luminal K^+^ significantly reduced V_te_ by approximately 20 % from -42 ± 4 to -33 ± 3 mV (N = 5, ± S.E.M., P < 0.05) after an average exposure time of 8 minutes, but the color change of mcresol purple was the same as observed under control conditions (Figure 12).

**Figure 4.**
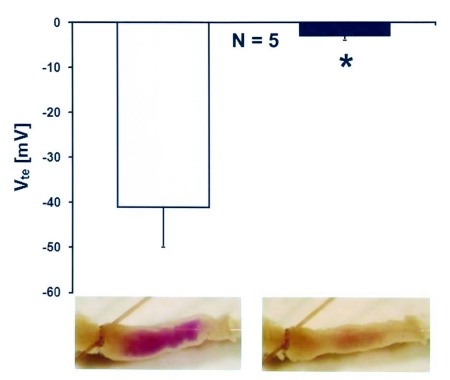
Mean lumen negative transepithelial voltage (V_te_; - S.E.M.) of 5 anterior midguts stimulated with serotonin (0.2 µmol l^-1^) in absence (white bar) and presence (black bar) of concanamycin (50 µmol l^-1^), and photographs of a representative preparation of the anterior midgut of larval (4^th^ instar) *Aedes* *aegypti* identical times after perfusion stop in presence (right) and absence (left) of concanamycin (50 µmol l^-1^). On the average the tissues were exposed for 60 minutes to concanamycin before V_te_ and alkalinization after the influence of the drug were recorded. Asterisk: Mean V_te_ significantly different from the control (P < 0.05).

### The magnitude of alkalinization

From the colors of m-cresol purple at different pH values (see Figure 1) it can be estimated that alkalinization in isolated anterior midgut preparations reached pH values above 9. In order to verify this estimate thymol blue was used. With a _p_K_2_ of 9.20, thymol blue exhibits a color change (from yellow to blue) at a higher pH value than m-cresol purple. In three experiments isolated anterior midgut preparations were perfused with low buffer saline containing 0.04 % thymol blue. After perfusion stop, color changes from yellow to blue were observed, confirming the above estimate that the alkalinization in isolated anterior midgut preparations reaches pH values above 9. In three experiments a pH-sensitive microelectrode was inserted into the lumen through the open end of the preparation. After stopping the luminal perfusion, V_te_ decreased, m-cresol purple changed color to pink, and the recorded pH rapidly increased. In one experiment (see Figure 13) the pH reached a value slightly above 10. The mean of three recordings after luminal perfusion stop averaged 9.5 ± 0.3 (± S.E.M.).

**Figure 5.**
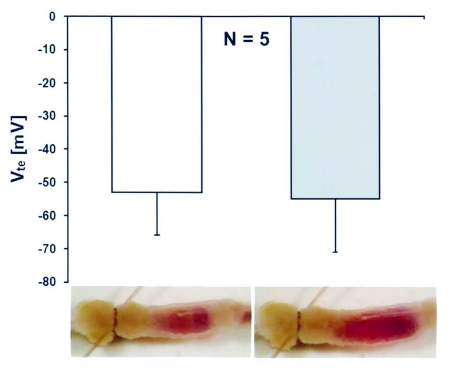
Mean lumen negative transepithelial voltages (V_te_; - S.E.M.) of 5 anterior midguts stimulated with serotonin (0.2 µmol l^-1^) in presence (grey bar) and absence (white bar) of hemolymph-side methazolamide (200 µmol l^-1^), and photographs of a representative preparation of the anterior midgut of larval (4^th^ instar) *Aedes* *aegypti* identical times after perfusion stop in presence (right) and absence (left) of hemolymph-side methazolamide (200 µmol l^-1^). Before V_te_ and alkalinization after the influence of the drug were recorded, the tissues were exposed 10–20 minutes to hemolymph-side methazolamide.

## Discussion

### Isolated midgut preparations lose the capacity for strong alkalinization

One problem that impedes analyses of the mechanisms of strong luminal alkalinization in isolated insect midgut regions is that *in vitro* preparations lose the capacity to generate high luminal pH values. Whereas living tobacco hornworms (*Manduca sexta*) were observed to generate a midgut pH of up to 12 (Dow 1984), the pH within the apical folds of the isolated tissue was only 7.8 (Dow and O'Donnell 1990). Indeed, acid-base fluxes have been measured with the isolated midgut of *M. sexta* (Chamberlin 1990; Coddington and Chamberlin 1999). However, the rates of these fluxes seem to be only a small fraction of the transport rates *in vivo* (Clark et al. 1998; Coddington and Chamberlin 1999). So far, strong luminal alkalinization in the anterior midgut of larval mosquitoes was only measured *in vivo* (Dadd 1975; Zhuang et al. 1999; Boudko et al. 2001a). The electrophysiological results with isolated and perfused anterior midguts of larval *A. aegypti* showed that the lumen negative, transepithelial voltage (V_te_) dramatically dropped to less negative values after mounting of the isolated tissue (Clark et al. 1999). With regard to the maintenance of the capacity of strong alkalinization in the *in vitro* preparation this observation was of course disappointing. Indeed, in the present study the color change of m-cresol purple observed in the isolated tissue after luminal perfusion stop was absent or weak (see Figure 3). Thus, as observed with isolated midgut epithelia of *M. sexta* (see above), the anterior midgut of larval *A. aegypti* loses at least a significant part of its capacity for strong alkalinization after isolation and mounting.

**Figure 6.**
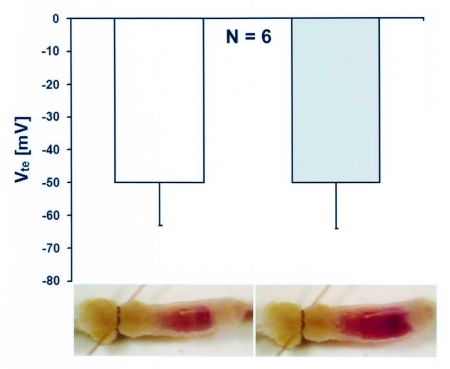
Mean lumen negative transepithelial voltages (V_te_; - S.E.M.) of 6 anterior midguts stimulated with serotonin (0.2 µmol l^-1^) in presence (grey bar) and absence (white bar) of luminal methazolamide (200 µmol l^-1^), and photographs of a representative preparation of the anterior midgut of larval (4^th^ instar) *Aedes* *aegypti* identical times after perfusion stop in presence (right) and absence (left) of luminal methazolamide (200 µmol l^-1^). Before V_te_ and alkalinization after the influence of methazolamide were recorded, the tissues were exposed 10–15 minutes to the drug in the luminal perfusate.

### Serotonin recovers strong alkalinization in larval mosquitoes

Isolated preparations of tobacco hornworm midgut were subjected to many treatments in order to stimulate acidbase transport to its *in vivo* rates (Clark et al. 1998). However, none of these manipulations re-established strong alkalinization. Serotonin, a possible transport stimulator that is present in *A. aegypti* hemolymph at submicromolar concentrations (Clark and Bradley 1997), only partly re-established the high lumen negative V_te_ that was measured with isolated anterior midguts of larval *A. aegypti* directly after mounting (Clark et al. 1999). After characterizing the V_te_ in the presence of serotonin as an expresssion of active HCO_3_^-^ secretion (Onken et al. 2004a), we assumed that serotonin only re-established HCO_3_^-^ secretion, but not the active transepithelial H^+^ absorption (or base secretion) necessary for strong alkalinization. Other attempts to restore the *in vitro* transepithelial potential difference with a number of peptide hormones (Onken et al. 2004b) were also unsuccessful, so we were very surprised when we observed in the present study that the isolated tissue generated strong alkalinization in the presence of serotonin only (see Figure 3, the controls of Figures 4–12, Video 1). Although the clearly pink color of m-cresol purple monitored in the present study can only be observed at pH values clearly higher than those that can be attributed to HCO_3_^-^ secretion (pH 8.5; cf. Figure 1), the high pH was verified using thymol blue (see Results) and pH sensitive microelectrodes (see Figure 13). The observed decrease of lumen negative V_te_ to less negative values that accompanied the growing alkalinization after luminal perfusion stop (see Figures 2 and 13) is difficult to interpret on the basis of the available data. Onken et al. (2004a) proposed that V_te_ reflects active HCO_3_^-^ secretion. Following this idea, it could be that an alkaline lumen stops or reduces HCO_3_^-^ secretion. This would make sense, because at a very alkaline luminal pH, HCO_3_^-^ secretion would reintroduce H^+^ into the lumen and decrease the pH. It could, however, also be that the mechanism of strong alkalinization itself is electrogenic, and that growing luminal alkalinization slows the process down until the set point pH is reached. This aspect needs to be analyzed in future studies in greater detail.

**Figure 7.**
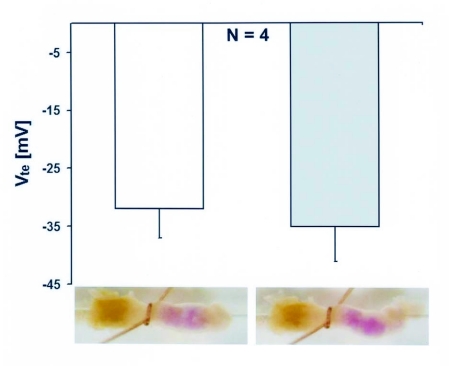
Mean lumen negative transepithelial voltages (V_te_; - S.E.M.) of 4 anterior midguts stimulated with serotonin (0.2 µmol l^-1^) in presence (grey bar) and absence (white bar) of luminal DIDS (1 mmol l^-1^), and photographs of a representative preparation of the anterior midgut of larval (4^th^ instar) *Aedes* *aegypti* identical times after perfusion stop in presence (right) and absence (left) of luminal DIDS. Before V_te_ and alkalinization after the influence of DIDS were recorded, the tissues were exposed 10–30 minutes to the drug in the perfusate.

Now that it is known that addition of serotonin is sufficient to re-establish strong alkalinization, the question of the discrepancy between the initially high V_te_ and the voltage recorded under perfusion with serotonin must be addressed. It may be that there is no elusive transport process that requires support of another modulator, but rather that the consequence of perfusing the gut interior with the same solution used in the bath eliminates diffusion potentials. Such a gradient was indeed measured in intact larvae where luminal Cl^-^ was low (3.5 mmol l^-1^) at high hemolymph Cl^-^ (58 mmol l^-1^; Boudko et al. 2001a). Thus, it could well be that the voltage decrease after mounting is partly based on elimination of a Cl^-1^ diffusion potential. Subjecting the isolated epithelium to a Cl^-1^ gradient by substitution of luminal Cl^-1^, did indeed result in a more negative V_te_ (see below). In any case, this work has established that serotonin alone is sufficient to reestablish alkalinization similar to that seen in the intact animal.

**Figure 8.**
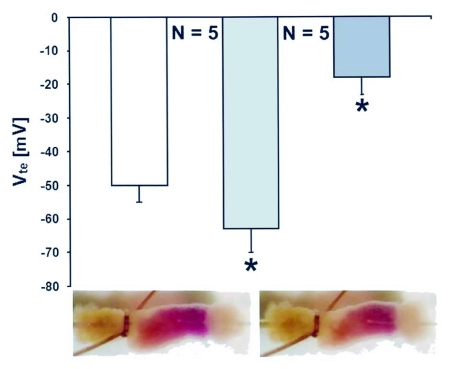
Mean lumen negative transepithelial voltages (V_te_; - S.E.M.) of 5 anterior midguts stimulated with serotonin (0.2 µmol l^-1^) before (white bar) and after substitution of Cl^-^ in the lumen (light grey bar) and on both sides of the tissue (dark grey bar), and photographs of a representative preparation of the anterior midgut of larval (4^th^ instar) *Aedes aegypti* identical times after perfusion stop in presence (left) and absence (right) of Cl^-^ on both sides of the tissue. Before V_te_ and alkalinization after both manipulations were recorded, the tissues were exposed to the new condition for an average of 10 minutes. Asterisk: Mean V_te_ significantly different from the control (P < 0.05).

### Strong alkalinization in larval mosquitoes: Involvement of basolateral V-ATPase

Based on observations with intact and semi-intact mosquito larvae it has been proposed that strong alkalinization is driven by V-type H^+^ pumps (Zhuang et al. 1999; Boudko et al. 2001 a, b). The proposal of active pumping of acid equivalents from the epithelial cells to the hemolymph is intuitively plausible for luminal alkalinization. In addition to inhibiting the transepithelial voltage (Onken et al. 2004a; this paper) and the transepithelial short-circuit current (Onken et al. 2006), hemolymphside concanamycin also inhibited the color change of mcresol purple, indicating blockage of alkalinization in the lumen of the isolated and perfused anterior midgut (see Figure 4). Because concanamycin is a specific inhibitor of V-ATPases (Dröse and Altendorf 1997), this result seems to confirm that the basolateral V-ATPase is of prominent importance for strong alkalinization. Indeed, the concentration of concanamycin used in the present study is much higher than normally used in biochemical studies with membrane vesicles. However, it has been observed before with the same and other inhibitors that higher concentrations of drugs are needed in intact cells and tissues. For example, fluid secretion in isolated Malpighian tubules is inhibited by 60 % after 40 minutes of incubation with 10 µmol l^-1^ bafilomycin (Beyenbach et al. 2000). Our study of isolated anterior midguts has a parallel with the complete inhibition of fluid secretion in Malpighian tubules, which was only observed at a concentration of 50 µmol l^-1^. The difference in susceptibility to certain drugs between biochemical and physiological assays may be related to a reduced accessibility of a drug to its target in the intact tissue.

**Figure 9.**
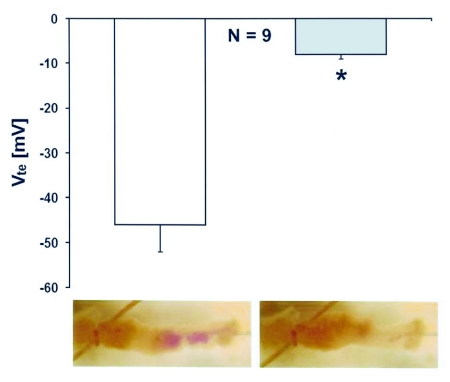
Mean lumen negative transepithelial voltages (V_te_; - S.E.M.) of 9 anterior midguts stimulated with serotonin (0.2 µmol l^-1^) before (white bar) and after (grey bar) substituting hemolymph-side Na^+^ and photographs of a representative preparation of the anterior midgut of larval (4^th^ instar) *Aedes aegypti* identical times after perfusion stop in presence (left) and absence (right) of Na^+^ in the hemolymph-side saline. The tissues were exposed for an average of 30 minutes to Na^+^-free saline before V_te_ and alkalinization after the influence of the manipulation were recorded. Asterisk: Mean V_te_ significantly different from the control (P < 0.05).

### Strong alkalinization in larval mosquitoes: Involvement of carbonic anhydrase

In intact mosquito larvae alkalinization was inhibited with acetazolamide/methazolamide, and in semi-intact larvae the drugs reduced the pH gradient at the hemolymph-side surface of the exposed anterior midgut. These results suggested that carbonic anhydrase (CA) is essential for strong alkalinization in the midgut of larval mosquitoes (Boudko et al. 2001a, Corena et al. 2002). Indeed, CA is of importance for many epithelial transport processes (Henry 1996) and its involvement in epithelial acid-base transport appears especially evident. CA is abundant in the anterior midgut of *M. sexta* (Ridgway and Moffett 1986), but CA inhibitors did not affect the remainder of the acid-base fluxes of the isolated epithelium (Coddington and Chamberlin 1999). In the isolated anterior midgut of larval *A. aegypti*, neither acetazolamide (see Onken et al. 2004a) nor methazolamide (see Figures 5, 6) significantly reduced V_te_ in the presence of serotonin. Moreover, also luminal alkalinization was unaffected by methazolamide in the luminal or hemolymp-side saline (see Figures 5, 6). We therefore conclude that strong alkalinization in the anterior midgut of larval *A. aegypti* is independent of CA, and we hypothesize that the inhibition of alkalinization by drugs implicated in CA inhibition in intact and semi-intact larvae is an effect of the drugs on systems that only indirectly interfere with alkalinization. This interpretation is consistent with the finding that CA activity is absent or very low in the anterior midgut of larval *A. aegypti* (Corena et al. 2002). Moreover, Hanson's histochemistry detected CA in epithelial cells of caeca and posterior midgut, but not in epithelial cells of the anterior midgut (Corena et al. 2002). On the other hand, CA IV was detected on the cell surface in the muscle network around the midgut, including the anterior midgut, and in nervous tissue, including nerves attached to the anterior midgut (Seron et al. 2004). As in the mammalian brain (cf. Kraig et al. 1983; Tong et al. 2000) cell surface CA IV could be involved in extracellular buffering and, thus, in the protection of muscles and nerves from the acidic microenvironment generated by transbasolateral acid flux via the neighboring epithelial cells in the anterior midgut. The inhibitory effect of acetazolamide/methazolamide on strong alkalinization in intact and semi-intact mosquito larvae might possibly be explained by inhibition of the surface buffering of serotonergic neurons (Moffett and Moffett 2005) and a subsequent decrease of serotonin liberation. In any case, the strong alkalinization of the isolated anterior midgut in the presence of methazolamide (Figures 5, 6) shows that carbonic anhydrase is not directly involved in the epithelial acid-base transport processes that result in very high luminal pH values.

**Figure 10.**
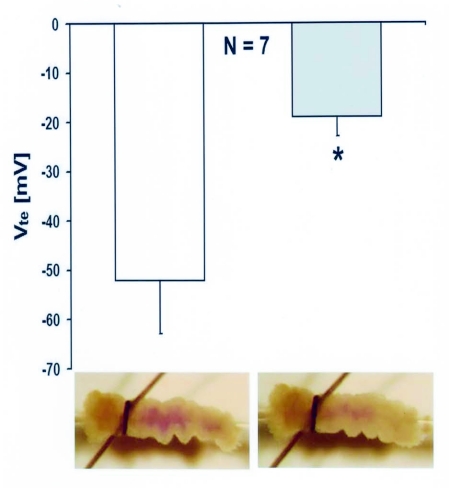
Mean lumen negative transepithelial voltages (V_te_; - S.E.M.) of 7 anterior midguts stimulated with serotonin (0.2 µmol l^-1^) in presence (grey bar) and absence (white bar) of hemolymp-side amiloride (200 µmol l^-1^), and photographs of a representative preparation of the anterior midgut of larval (4^th^ instar) *Aedes* *aegypti* identical times after perfusion stop in presence (right) and absence (left) of hemolymph-side amiloride (200 µmol l^-1^). Before recording V_te_ and alkalinization after amiloride the tissues were exposed to the drug for an average time of 25 minutes. Asterisk: Mean V_te_ significantly different from the control (P < 0.05).

**Figure 11.**
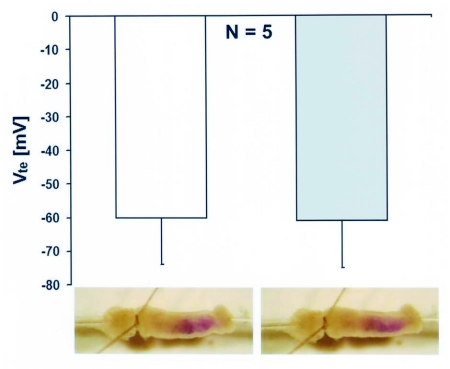
Mean lumen negative transepithelial voltages (V_te_; - S.E.M.) of 5 anterior midguts stimulated with serotonin (0.2 µmol l^-1^) in presence (grey bar) and absence (white bar) of luminal amiloride (200 µmol l^-1^), and photographs of a representative preparation of the anterior midgut of larval (4^th^ instar) *Aedes* *aegypti* identical times after perfusion stop in presence (right) and absence (left) of luminal amiloride (200 µmol l^-1^). Before recording V_te_ and alkalinization after amiloride the tissues were exposed to the drug for 10–30 minutes.

### Strong alkalinization in larval mosquitoes: Involvement of HCO_3_^-^ secretion

Active HCO_3_^-^ secretion alone cannot generate a strong alkalinization above pH values of 8.5, and therefore cannot offer a complete explanation for alkalinization. However, evidence for HCO_3_^-^ secretion is provided by *in vitro* measurements: from the HCO_3_^-^/CO_3_^2^- concentrations measured in the hemolymph and in the anterior midgut lumen of larval *A. aegypti* (4 and 58 mmol l^-1^, respectively; Boudko et al. 2001a) the presence of active HCO_3_^-^ secretion seems evident, and the reversed Cl^-^ concentrations (3.5 mmol l^-1^ in the lumen and 58 mmol l^-1^ in the hemolymph; Boudko et al. 2001a) suggest that this secretion follows a transepithelial Cl^-^/HCO_3_^-^exchange mechanism. It could therefore be that active HCO_3_^-^ secretion is a prerequisite for strong alkalinization. Indeed, m-cresol purple in the anterior midgut of intact larvae changed from purple to yellow when larvae were exposed to 0.1 mmol l^-1^ DIDS in the medium (Boudko et al. 2001a). This finding resulted in the proposal that HCO_3_^-^ secretion via apical anion exchangers is essential for strong alkalinization. However, in the isolated preparation, luminal DIDS did not affect the color change of m-cresol purple after perfusion stop even at a much higher concentration than used with intact larvae (see Figure 7). Moreover, even substitution of luminal and hemolymph-side Cl^-^ did not affect strong alkalinization (see Figure 8). These results seem to rule out HCO_3_^-^ secretion via apical Cl^-^/HCO_3_^-^ exchangers as a component of strong luminal alkalinization.

**Figure 12.**
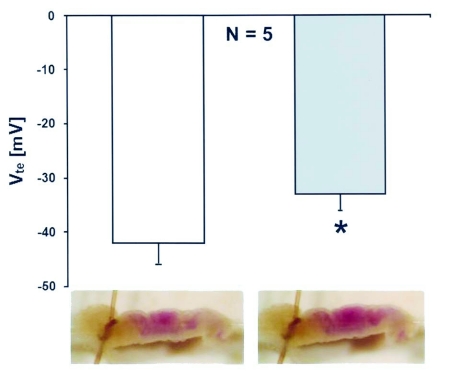
Mean lumen negative transepithelial voltages (V_te_; - S.E.M., N = 5) of 5 anterior midguts stimulated with serotonin (0.2 µmol l^-1^) before (white bar) and after (grey bar) increasing luminal K^+^ to 60 mmol l^-1^, and photographs of a representative preparation of the anterior midgut of larval (4^th^ instar) *Aedes* *aegypti* identical times after perfusion stop in presence (right) and absence (left) of high K^+^ in the luminal perfusion saline. Before recording V_te_ and alkalinization in the presence of increased luminal K^+^ the tissues were exposed to this condition for an average time of 8 minutes. Asterisk: Mean V_te_ significantly different from the control (P < 0.05).

Consequently, the possibility that the effects of DIDS on midgut alkalinization in intact larvae were indirect must be considered. DIDS is a rather unspecific inhibitor, interacting with many different transporters, including anion exchangers, anion channels, KCl cotransporters and Na^+^/HCO_3_^-^ cotransporters (cf. Culliford et al. 2003; Boron 2001; Reddy and Quinton 2002). When working with intact larvae it cannot be excluded that DIDS enters into the hemolymph before the drug even reaches the anterior midgut. From the hemolymph the drug could interfere with many cells and tissues, indirectly resulting in the impairment of midgut alkalinization.

In a previous study, we reported somewhat different observations on the effects of DIDS and Cl^-^ substitution on V_te_. Onken et al. (2004a) measured a two step V_te_ decrease to less negative values when DIDS (0.1 mmol l^-1^) was added first to the hemolymph-side bath and then introduced into the lumen by changing the pump from infusion to withdrawal. In the present study, a higher concentration of DIDS (1 mmol l^-1^) directly infused into the lumen did not at all affect V_te_. Because changing the pump direction can cause voltage artifacts (cf. Onken et al. 2004a) this methodological difference cannot be excluded as the source for the different findings with DIDS. However, the possibility that an effect of luminal DIDS on V_te_ becomes only detectable after prior inhibition of a basolateral anion transporter also cannot be excluded. Onken et al. (2004a) also found that Cl^-^ substitution in the hemolymph-side bath followed by Cl^-^ substitution on both sides of the tissue caused a two step V_te_ decrease to less negative values (Onken et al. 2004a). In the present study, luminal Cl^-^ substitution increased lumen negative V_te_ to more negative values and only bilateral Cl^-^ substitution decreased it. After observing the different response of V_te_ during Cl^-^ substitution experiments, we performed an experiment in which we used the same sequence as in Onken et al. (2004a; first hemolymph-side and then bilateral Cl^-^ substitution) and obtained the same result as in the previous paper. Consequently, we conclude that a different sequence of Cl^-^ substitution (first hemolymph-side and then bilateral or first luminal and then bilateral) results in different responses of V_te_. The differences between the results in the present and previous study are difficult to interprete and need to be addressed in the future.

**Figure 13.**
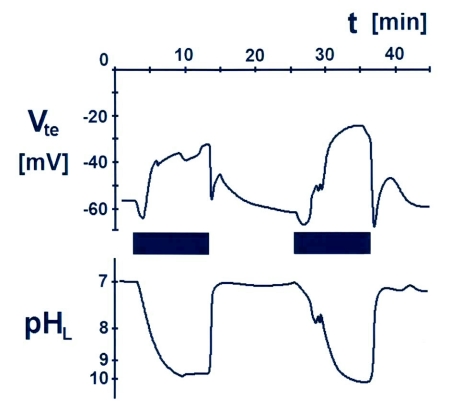
Time-course of the lumen negative transepithelial voltage (V_te_) and of the luminal pH (_p_H_L_) recorded with a pH-sensitive microelectrode in the lumen of a preparation of the anterior midgut of larval (4^th^ instar) *Aedes aegypti*, showing the effects of repeated luminal perfusion stops in the presence of hemolymph-side serotonin (0.2 µmol l^-1^).

In a previous study (Onken et al. 2004a), we found that hemolymph-side amiloride or substitution of Na^+^ markedly reduced lumen negative V_te_ to less negative values and it was proposed that a part of active, transepithelial base secretion/acid absorption proceeds via basolateral Na^+^/H^+^ exchangers and apical Na^+^/2-3HCO_3_^-^ symporters. In the present study (see Figures 9, 10) we confirmed the effects of these manipulations on V_te_. In addition, hemolymph-side amiloride and Na^+^ substitution clearly affected the color change of mcresol purple after perfusion stop, indicating impairment of strong alkalinization. In accordance with our interpretations from the previous paper, it could be concluded that the Na^+^ -dependent, transepithelial HCO_3_^-^ secretion is involved in strong luminal alkalinization of the anterior midgut of larval *A. aegypti.* However, the electrogenic Na^+^/HCO_3_^-^ symporters are usually sensitive to DIDS (cf. Boron 2001), which, in the present study, did not influence V_te_ or strong alkalinization (see above). Therefore, alternative interpretations for the effects of hemolymph-side amiloride and Na^+^ substitution must be considered. In addition to interfering with Na^+^/H^+^ exchange, amiloride and Na^+^ -free saline also inhibit Na^+^/Ca^2+^ exchangers (cf. Egger and Niggli 1999), resulting in an increase of cellular Ca^2+^. Thus, it could be that the strong alkalinization in the anterior midgut that appeared to be dependent on HCO_3_^-^ secretion, is actually regulated by intracellular Ca^2+^. This hypothesis should be tested with Ca^2+^ ionophores.

### Strong alkalinization in larval mosquitoes: Involvement of apical K^+^/2H^+^

For the midgut of larval *M. sexta* it was proposed that apical V-ATPases energize apical K^+^/2H^+^ antiport, resulting in active K^+^ secretion and strong luminal alkalinization (Wieczorek et al. 1991; Azuma et al. 1995). At a low luminal K^+^ concentration in the midgut lumen of larval *A. aegypti* an apical K^+^/2H^+^ exchanger could make use of a cell negative transapical voltage (cf. Clark et al. 2000) and of a transapical K^+^ concentration gradient to generate H^+^ absorption against a large gradient. 200 µmol l^-1^ amiloride inhibited 50 % of K^+^/2H^+^ antiport in membrane vesicles of *M. sexta* goblet membranes (Wieczorek et al. 1991). Perfusing the lumen of the anterior midgut of *A. aegypti* with this concentration of amiloride had no effect on V_te_ or the color change of m-cresol purple after perfusion stop (see Figure 11). However, in the isolated *M. sexta* midgut epithelium, amiloride affected the transapical short-circuit current only at 10 mmol l^-1^ (Schirrmanns and Zeiske 1994), a concentration of the drug that also inhibits the V-ATPase itself (Wieczorek et al. 1991). As an alternative test for the hypothesis that strong alkalinization in larval *A. aegypti* is based on apical K^+^/2H^+^ we increased the luminal K^+^ concentration to 60 mmol l^-1^. This concentration is close to the intracellular K^+^ concentration of epithelial cells from the Malpighian tubules of *A. aegypti* (75 mmol l^-1^, Petzel et al. 1999) and should, thus, markedly reduce the transapical K^+^ concentration gradient. However, in the presence of high K^+^ in the lumen the color change of m-cresol purple was observed as under control conditions (see Figure 12). Based on these findings with amiloride and high K^+^ in the luminal perfusate we propose that apical K^+^/2H^+^ antiport is not involved in strong alkalinization in the anterior midgut of larval *A. aegypti*. The 20 % reduction of V_te_ in the presence of high luminal K^+^ can be interpreted as reflecting different paracellular permeabilities for Na^+^ and K^+^. The V_te_ reduction would then indicate that the permeability of the paracellular junctions is higher for Na^+^ than for K^+^.

### Final remarks

In summary, the present study shows that the isolated and perfused anterior midgut of larval *A. aegypti* generated strong alkalinization in the presence of serotonin. The technique employed determines whether alkalinization is present under a certain condition, but it has the disadvantage that acid-base fluxes cannot be quantified. However, measurements with pH-sensitive microelectrodes (see Figure 13) confirmed the magnitude of alkalinization. Assuming that alkalinization is based on H^+^ absorption, the results shown in Figure 13, the physical dimensions of the anterior midgut given in Clark et al. (2000), and the amount of base needed to titrate a volume of low buffer saline from pH 7 to pH 10 (30 mequiv l^-1^; mainly reflecting the buffer capacity of the amino acids of the saline) can be used to estimate a flux of 1–2 µmol cm^-2^ h^-1^. Indeed, this value is markedly smaller than the fluxes obtained with isolated midgut preparations of larval *M. sexta*, which were considered much lower than *in vivo.* However, it has to be considered that the ratio of epithelial surface and midgut volume is much larger in the small midgut of larval mosquitoes. In other words, in larval mosquitoes a much smaller surface-specific H^+^ absorption is necessary to increase the pH in the much smaller midgut volume.

The results of the present study indicate that, with the exception of V-ATPase involvement, all previous proposals about the mechanisms of strong alkalinization in larval mosquitoes seem to not apply. Indeed, our results do not exclude the presence of active HCO_3_^-^ secretion, but they indicate that this process is not a prerequisite for strong alkalinization. Of course, on first sight our results seem to be disappointing. On the other hand, the observation that an *in vitro* preparation maintains its capacity for strong midgut alkalinization is an essential breakthrough. The experimental advantages of isolated epithelia for the analysis of strong alkalinization in an insect midgut suggest that understanding the underlying mechanisms can be achieved in the near future.
